# A Critical Review of Proteomic Studies in Gestational Diabetes Mellitus

**DOI:** 10.1155/2020/6450352

**Published:** 2020-07-14

**Authors:** Tao Zhou, Lu Huang, Min Wang, Daozhen Chen, Zhong Chen, Shi-Wen Jiang

**Affiliations:** ^1^Research Institute for Reproductive Medicine and Genetic Diseases, The Affiliated Wuxi Maternity and Child Health Care Hospital of Nanjing Medical University, Wuxi 214002, China; ^2^Department of Obstetrics, The Affiliated Wuxi Maternity and Child Health Care Hospital of Nanjing Medical University, Wuxi 214002, China; ^3^Centre for Reproductive Medicine, The Affiliated Wuxi Maternity and Child Health Care Hospital of Nanjing Medical University, Wuxi 214002, China

## Abstract

Gestational diabetes mellitus is a progressive and complex pregnancy complication, which threatens both maternal and fetal health. It is urgent to screen for specific biomarkers for early diagnosis and precise treatment, as well as to identify key moleculars to better understand the pathogenic mechanisms. In the present review, we comprehensively summarized recent studies of gestational diabetes using mass spectrometry-based proteomic technologies. Focused on the entire experimental design and proteomic results, we showed that these studies have covered a broad range of research contents in terms of sampling time, sample types, and outcome associations. Although most of the studies only stayed in the stage of initial discovery, several proteins were further verified to be efficient for disease diagnosis. Functional analysis of all the combined significant proteins also showed that a small number of proteins are known to be involved in the regulation of insulin or indirect signaling pathways. However, many factors such as diagnostic criteria, sample processing, proteomic method, and statistical method can greatly affect the identification of reproducible and reliable protein candidates. Thus, we further provided constructive suggestions and recommendations for carrying out proteomic or follow-up studies of gestational diabetes or other pregnancy complications in the future.

## 1. Introduction

Gestational diabetes mellitus (GDM) is currently defined as a separate subcategory of diabetes, which is only accompanied by pregnancy [1, 2]. It is known that the high levels of hormones, such as progesterone and placental lactogen, may promote insulin resistance and result in hyperglycemia during pregnancy [3]. There are many pathogenic risk factors for GDM, including high body mass index (BMI), advanced maternal age, and family history of diabetes. Partially due to the boom of elderly parturient women and the transition of modern lifestyle, the prevalence of GDM was reported to be increasing over the past decades [4, 5]. Large cohort studies indicated that GDM is associated with various pregnancy and delivery complications such as preeclampsia (PE), caesarean delivery, macrosomia, neonatal hypoglycemia, and large for gestational age [6–8]. Exposure to hyperglycemia during pregnancy may also result in long-term adverse effects on both postpartum women and newborns. For example, women with GDM were found to have an elevated risk of developing type 2 diabetes (T2D) and cardiovascular diseases [9, 10]. The offsprings may also be at a high risk of suffering from diabetes, obesity, hypertension, and dyslipidemia later on in their life [11]. Moreover, current treatments may have limited effects on early GDM (diagnosed in high-risk women at <24 weeks of gestation) [12]. A recent study also showed that the incidences of neonatal hypoglycemia and polyhydramnios remain high after insulin treatment [13]. Thus, GDM has become a serious public health problem that increases health care costs during and post pregnancy [14].

There are several versions of screening and diagnostic criteria for GDM, as recommended by the International Association of the Diabetes and Pregnancy Study Groups (IADPSG) [15], the American Diabetes Association (ADA) [1], or the World Health Organization (WHO) [16]. Although the details of different criteria are frequently revised and still under debate, some common guidelines are accepted based on the Hyperglycemia and Adverse Pregnancy Outcome (HAPO) studies [17, 18]. First, GDM screening is usually suggested to be performed at 24-28 weeks of gestation (the second trimester), leaving only limited time for interventions or treatments. Second, laboratory measurements for traditional diabetes such as random plasma glucose (RPG), fasting plasma glucose (FPG), or hemoglobin A1c (HbA1c) are not directly applicable for GDM. Instead, one-step or two-step approaches based on the oral glucose tolerance test (OGTT) are widely used. However, there exists a portion of false detections and lacks precise cutoffs for predicting the outcomes using the current tests. Furthermore, most standards suggested to distinguish and diagnose overt diabetes or pre-existing pre-gestational diabetes in the early stage of pregnancy before the diagnosis of GDM. Hence, it is of vital importance to screen for specific biomarkers for early detection, differential diagnosis, outcome prognosis, and precise treatment of GDM, as well as to identify key moleculars to better understand the basic mechanism of pathogenesis.

In clinical research, a biomarker is defined as any substance (such as RNA, proteins, or metabolites) that could be measured for the prediction of the incidence or outcome of a disease [19]. The predictive efficiency of a biomarker is evaluated by the area under the curve (AUC), as calculated based on the paired values of sensitivity and specificity for a biomarker. In general, a prediction model is considered to be acceptable when the AUC value is above 0.6 [20]. An AUC larger than 0.8 indicates a very good accuracy, while a value greater than 0.9 indicates an excellent diagnostic accuracy. As the major players of gene functions, proteins presented in various types of body fluids or tissues have been a focus for searching specific and sensitive disease biomarkers. Since GDM is believed to share some common pathogenic mechanisms with T2D, several studies have tested and evaluated a selection of protein biomarkers related to plasma glucose, insulin resistance, inflammatory pathway, or oxidative stress [21–23]. However, few proteins have shown to be efficient for the prediction of impending gestational diabetes and some results are still needed to be validated. Over the past decades, proteomic methods based on mass spectrometry (MS) are rapidly developing and have been applied in various fields of biomedical researches. Basically, MS-based proteomics can identify and quantify various proteins precisely based on the mass and intensity of fragmented peptides [24]. The development of biomarkers using a proteomic approach usually undergo three successive stages including discovery, verification, and validation, with an increasing number of enrolled subjects and a decreasing number of candidate biomarkers [25]. One of the main advantages of proteomic approaches is that it can systematically evaluate the expression of the whole proteins of multiple samples in a single experiment. In addition, the recently developed targeted proteomic strategies, which do not require any antibodies, could replace traditional western blot or enzyme-linked immunosorbent assay (ELISA) as the new golden standard for protein verification and validation [26].

In recent years, more and more proteomic studies have been performed to construct differential expression profiling or to identify potential biomarkers for GDM and its complications. After an extensive PubMed search for proteomic studies of GDM using mass spectrometry, a total of 21 representative full-length research articles are included in this review [27–47]. The detailed information of the reviewed literatures are listed in Supplementary Data 1. Focused on the entire experimental design and proteomic results, the present review comprehensively summarizes and compares these studies. All of the datasets are also integrated to obtain an overview of functional pathways for those differentially expressed (DE) proteins between different groups. Finally, the limitations of current studies and possible improvements toward translational and precision proteomics are also discussed in depth.

## 2. Summary of Proteomic Studies of GDM

### 2.1. Experimental Designs and Objectives

**Figure 1:**
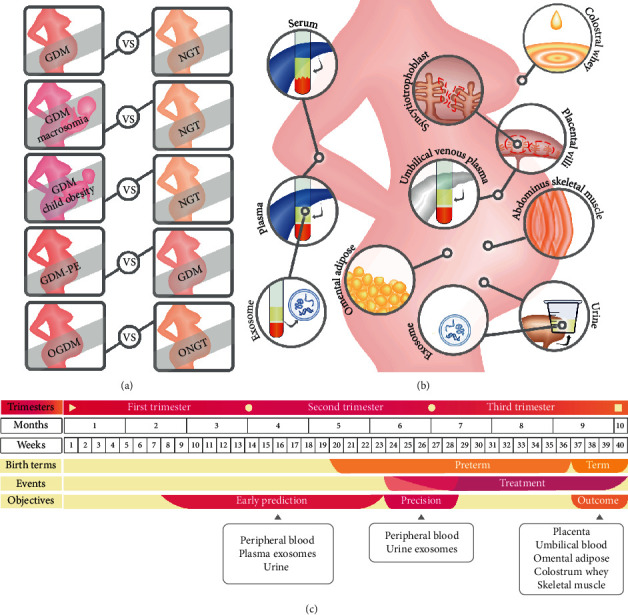
Summary of the experimental designs applied in the reviewed studies. (a) Five pairs of disease-control design for GDM and its adverse effects; (b) eleven types of samples for proteomic analyses; (c) a summarized timetable of pregnancy trimesters (the whole pregnancy period is divided into three trimesters), pregnancy time (in months or weeks), birth terms (preterm or term birth), clinical events for GDM (diagnosis time or treatment time), potential research objectives of these proteomic studies (early prediction, precision diagnosis, or outcome prognosis), and sample types.

In order to identify or quantify important proteins for GDM, most studies adopted a traditional disease-control design. Thus, GDM cases were usually treated as disease groups, while those cases with normal glucose tolerance (NGT) were regarded as control groups (Figure 1(a)). However, two studies are aimed at investigating adverse fetal outcomes, which selected GDM with macrosomia [33] or GDM with childhood obesity [35] as disease groups. Another study intended to identify biomarkers for one of the GDM-associated complications (early-onset PE) [40], which selected GDM as control groups. Additionally, one study is aimed at comparing GDM with NGT in obese pregnant women [43].

The research materials and sampling time further determine the research objectives. In clinical studies, the peripheral circulating blood is the most widely used specimen for searching biomarkers. Plasma or serum components can further be extracted from blood to remove cell parts or platelets. Among the ten studies using peripheral blood, five studies used plasma specimens (Figure 1(b)) [27, 32, 39, 44, 47], and the remaining studies used serum samples. Moreover, there is also a growing trend for seeking noninvasive testing strategies nowadays. Hence, one study used urine samples to identify biomarkers that could predict GDM [38].

In recent years, increasing attention is drawn to the utility of exosomes in monitoring various diseases. The concentration of circulating exosomes is found to be elevated in GDM cases [48]. Since circulating exosomes could be originated from the placenta and deliver to various types of cells, it is a novel and promising approach to elucidate the physiological mechanisms of GDM based on the proteins identified in the isolated plasma exosomes [41] or even urine exosomes [45]. It is generally accepted that the basic pathogenesis of GDM is insulin resistance. Thus, one study used omental adipose tissue, which is known to play important roles in metabolic disorders, to screen for key proteins involved in pregnancy-induced insulin resistance [34]. In addition, abdominus skeletal muscle tissue was used to investigate the metabolic consequences of GDM in obese pregnant women [43].

GDM is a complex complication affecting both maternal and fetal health. Several studies used specimens associated with fetomaternal communication, such as placental tissue (villi) [28, 36], syncytiotrophoblast [40], and umbilical venous plasma [33, 35, 39, 44]. These samples could provide key proteins for understanding the pathogenesis and progression of GDM, especially for GDM-related maternal complications and adverse fetal outcomes. In addition to the maternal-fetal interface, breast milk delivers nutrition to breastfed infants. Thus, one study showed that colostral whey proteins involved in immunity and nutrition are significantly changed in GDM [30].

Pregnancy is a progressive physiological process and lasts about 40 weeks. The gestational period is usually divided into three sections (trimesters). Each trimester lasts about three months or 13 weeks. As most of the hormones with diabetogenic potency were found to reach a peak around 24-28 weeks during pregnancy [49], the recent versions of guides (such as IADPSG 2010, WHO 2013, and ADA 2019) all recommended to perform screening and diagnosis of GDM at 24-28 weeks of gestation. After diagnosis, GDM patients are usually treated by dietary control or insulin intervention to maintain their fasting plasma glucose levels. There are generally three periods of sampling time among the reviewed studies (Figure 1(c)). First, peripheral blood and urine samples were drawn before diagnosis and used to identify biomarkers for early prediction [27, 32, 37, 38, 42, 47]. Second, peripheral blood and urine exosome samples were also drawn at the same time with diagnosis and used to identify biomarkers for precision diagnosis or classification [29, 31, 41, 45]. Third, placental tissues, umbilical blood, omental adipose tissues, abdominus skeletal muscle tissue, and colostrum whey were obtained during or after delivery and used to identify markers for the pathogenesis of GDM or fetal outcomes [28, 30, 33–36, 39, 40, 43, 44, 46].

### 2.2. General Experimental Workflow

**Figure 2:**
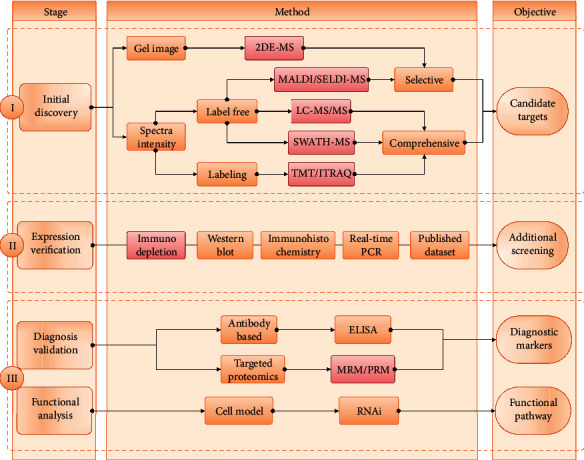
Schematic overview of proteomic-driven analyses of GDM. A summarized overview of stages, methods, and objectives for proteomic-driven analyses of GDM. Method boxes with a red border indicate the involvement of mass spectrometry.

By combining all the reviewed studies, the general workflow of the proteomic-based discovery of biomarkers for GDM is shown in Figure 2. In brief, the whole workflow can be divided into three stages: initial discovery, expression verification, and final validation (for diagnosis or functional analysis). In the first stage, a proteome-wide quantitative approach was applied to identify differentially expressed proteins or peptides between GDM and the control groups. There are generally two types of proteomic quantitative strategies, which are based on gel image or spectrum intensity (label free or chemical labeling) separately. We further classified these technologies into two groups based on their throughput and identification coverage: the selective and comprehensive strategies. The classic proteomic methods such as two-dimensional gel electrophoresis (2DE), matrix-assisted laser desorption/ionization (MALDI), or surface-enhanced laser desorption/ionization (SELDI) are seen as low-throughput and selective approaches. In these approaches, only several differentially expressed spots or spectra are usually selected for the follow-up mass spectrometry analysis, which generate a selective list of protein identifications. Nowadays, more and more studies used the state-of-art proteomic technology, known as liquid chromatography-tandem mass spectrometry (LC-MS/MS), to comprehensively identify and quantify all the proteins in the initial discovery step. These approaches first generate a full list of protein identifications; then, a sublist of differentially expressed proteins are further identified based on label-free or labeling information. Although labeling methods based on isobaric tags for relative and absolute quantitation (iTRAQ) or tandem mass tag (TMT) are believed to be more reliable in accuracy, label-free-based methods could identify more proteins and cover a broad range of expression levels [50]. A previous study also showed that a label-free-based proteomic quantification approach could be used to quantify plasma biomarkers in clinic with high efficiency [51]. The traditional LC-MS/MS is running in a data-dependent acquisition (DDA) mode, which only allows the most intense ions to be analyzed. In contrast, the data-independent acquisition (DIA) mode records all the fragmented spectra of the entire isolated ions within a certain window [52]. The recently prevalent sequential window acquisition of all theoretical mass spectra (SWATH) technology, a representative strategy of DIA, is believed to be a promising tool for biomarker discovery and translational proteomics [53]. Among the reviewed studies, five of them used the selective approaches based on 2DE, MALDI, or SELDI quantification; 15 studies used the comprehensive LC-MS/MS methods based on label-free, iTRAQ, or TMT quantification; and one study applied the SWATH-MS technology for label-free quantification.

All the reviewed studies performed an initial screening of potential biomarkers or functional proteins. However, a few studies stopped in the first stage without the follow-up verification or validation analyses. The general workflow for developing clinic biomarkers usually undergoes multiple rounds of verification or validation to obtain the final list of proteins with enough diagnostic capacity. ELISA (enzyme-linked immunosorbent assay) is a classic and powerful tool for validating protein biomarkers. However, the sensitivity and specificity of ELISA largely depended on the quality of the corresponding antibodies. The development and production of antibodies take a lot of time, money, and resource and also have a high rate of failure. The recently emerging targeted proteomic technologies, such as multiple reaction monitoring (MRM) or parallel reaction monitoring (PRM), can quantify multiple proteins in a single experiment, providing a promising alternative for biomarker validation [54]. Another advantage of targeted proteomics is that it can handle protein isoforms and post-translational modifications (PTM) at the same time. Among the reviewed studies, eight of them used the ELISA method, while one recent study applied the MRM technology to validate the expression of selected candidates.

Besides identifying diagnostic biomarkers, several studies are also aimed at identifying key proteins that are involved in the pathogenesis of GDM and its outcomes. However, most of the reviewed studies only discussed the potential functional associations of the identified DE proteins theoretically or based on bioinformatics annotations. Very few studies have performed follow-up functional experiments. For example, one study performed RNA interference (RNAi) experiment on cultured cells (adipocytes) to analyze the in vitro functions of adipocyte plasma membrane-associated protein (APMAP), one of the downregulated proteins in GDM groups, and found that APMAP may play an important role in the impaired insulin signaling pathway [34]. In addition to clinical validation or functional verification, eight studies also performed expression verification experiments using immunodepletion, western blot, immunohistochemistry, real-time polymerase chain reaction, or bioinformatics database (the Human Protein Atlas) [55] to verify the existence, localization, and change trend of DE proteins.

### 2.3. Diagnostic Efficiency of Protein Biomarkers

As shown in Table 1, five studies evaluated the diagnostic efficiency of a selected list of significantly changed proteins [31, 37, 38, 42, 47]. The concentrations of target proteins were measured by the ELISA or MRM method in serum, plasma, or urine samples. One of the advantages of proteomic-driven biomarker discovery is that a list of protein candidates could be easily used to establish a model with multiple indicators. Compared to a single protein biomarker, a combination of multiple protein biomarkers can greatly improve diagnostic accuracy. For example, the AUC was increased from 0.81 to 0.85 combining two glycosylated proteins (fibronectin-SNA and PSG-AAL) [31]. And an AUC of 0.97 can be reached further combining several biochemical indicators (CRP, adiponectin, SHBG, and ratio of hCG to placental lactogen) associated with GDM. Molecular biomarkers could also be used in conjunction with clinical characteristics to improve diagnosis. For example, vitronectin was validated to be significantly changed in GDM [42]. And the AUC was increased from 0.625 to 0.806 when vitronectin was used together with maternal age and history of diabetes.

It is also exciting to see several protein biomarkers that were identified in the early stages of pregnancy before GDM diagnosis. These markers could be used for early prediction of GDM, leaving enough time for clinical interventions to prevent later adverse outcomes. Currently, most biomarkers were detected in serum or plasma. However, CD59 and IL1RA were tested using urine samples, providing a noninvasive way of diagnosis. As most biomarkers were evaluated using a small sample size, further rounds of expression validation and statistical assessment are required before these biomarkers are being applied in clinic.

### 2.4. Functional Annotation of Significant Proteins

**Figure 3:**
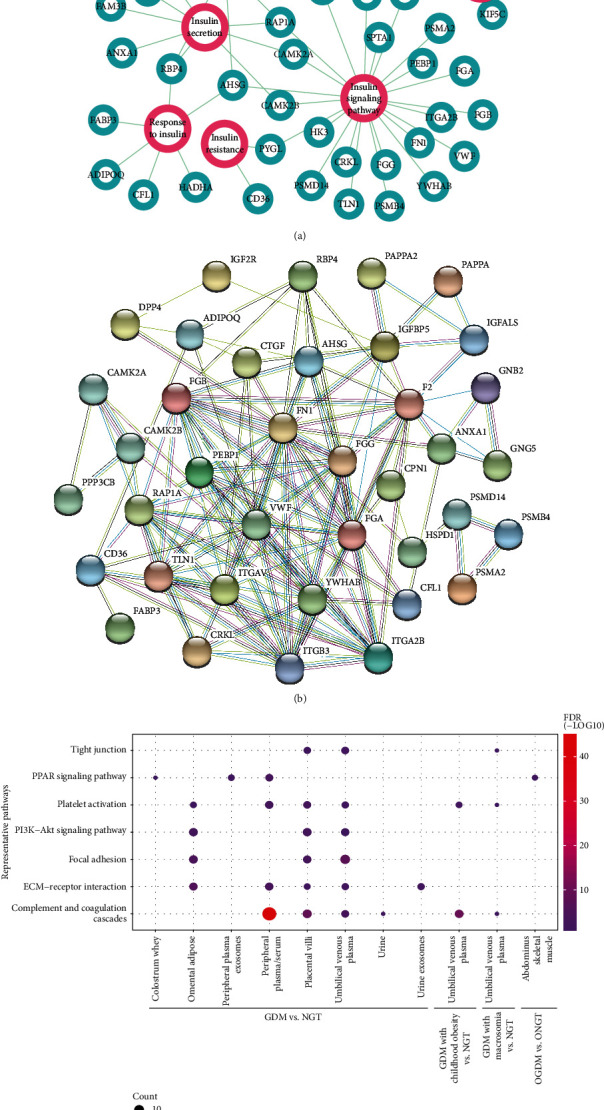
Functional annotation of the integrated differentially expressed proteins. (a) Relation network of differentially expressed proteins and insulin functions; (b) protein-protein relation network of insulin-associated DE genes; (c) representative enriched pathways for different groups of DE genes. Circle size is proportional to the number of gene count, while graduated color indicates the corresponding value of FDR.

In addition to identify potential biomarkers, the DE proteins could also help us to better understand the molecular mechanisms associated with the pathogenesis of GDM and its outcomes. To obtain a functional overview, we integrated and classified all the DE or validated proteins according to comparison design and sample type (Supplementary Data 2). The ToppGene suite was used for functional annotation based on Gene Ontology (GO) and Kyoto Encyclopedia of Genes and Genomes (KEGG) pathways [56]. First, we systematically searched for known markers related to insulin functions. Among the 417 integrated genes, 45 of them are found to be directly associated with insulin secretion, binding, response, regulation, resistance, or other signaling pathways (Figure 3(a)). We also found that 38 of these genes are highly associated with each other using the STRING database [57] (Figure 3(b)).

As mentioned above, the reviewed studies could be firstly divided into five classes based on the research object: GDM versus NGT, GDM versus GDM with PE (maternal complication), OGDM versus ONGT, GDM with macrosomia versus NGT (fetal outcome), and GDM with childhood obesity versus NGT (long-term fetal effect). And eleven types of samples were used in these studies: peripheral plasma/serum, peripheral plasma exosomes, placental villi, syncytiotrophoblast, umbilical venous plasma, urine, urine exosomes, omental adipose tissues, abdominus skeletal muscle tissues, and colostrum whey. We divided the dataset into 12 groups with a combination of experimental comparison and sample type. We thus performed functional enrichment analysis to identify overrepresented KEGG pathways in different groups, which may provide novel insights into the complex pathogenesis of GDM. A *P* value of 0.05 was used as a cutoff for statistical significance. Only about 11.5% of the integrated proteins were overlapped with each other among the total 12 groups. However, it is interesting to see many of the top enriched pathways are presented in more than one grouped class (Figure 3(c); Supplementary Data 3). Although the DE proteins are largely different among various samples or groups, the same enriched pathways indicated that common pathogenic mechanisms may be identified. Several pathways including complement and coagulation cascades, platelet activation, ECM-receptor interaction, PI3K-Akt signaling pathway, and PPAR signaling pathway, which are already known to be associated with insulin resistance or T2D [58–62], are still significantly enriched after the applying of false discovery rate (FDR) correction. The samples of these studies were obtained at different gestational ages. Focused on the DE proteins from peripheral plasma or serum, we also compared the enriched pathways identified in different sampling times. As expected, most of the enriched pathways in the combined peripheral blood proteins are repeatedly identified in the subclasses (Supplementary Data 4). And the top enriched pathways in different periods are slightly different. GDM is known to be a progressive disease; the differential distributions of the enriched pathways in different gestational weeks may reflect the corresponding stages of GDM. However, it still needs more experiments and data to prove and organize the results.

## 3. Bottlenecks and Limitations of Current Studies

### 3.1. Variation of Proteomic Methods and Experimental Design

As described above, although many studies focused on the differences between GDM and NGT, few proteins are found to be overlapped. In addition, from DE proteins to potential biomarkers, there are also a low proportion of targets that could pass the validation testing. For example, one study identified 25 DE proteins in serum, while six of them can be used to develop a testing assay and only one protein was proved to be effective in the final predictive model [42]. There are many factors that may contribute to the high rates of variations and low rates of validations. One major factor that may result in variations is the difference of proteomic methods. One study compared the gel-based and gel-free methods using the same placenta samples and found that only 5 proteins were overlapped in a total of 42 DE proteins [36]. Since various proteomic methods such as 2DE, MALDI, SELDI, LC-MS/MS, and SWATH were used to discover significant proteins in the reviewed studies, there may be random biases in the detection of DE proteins.

Actually, because the detailed experimental design and procedure vary in different researches, most studies are not directly comparable. However, currently, there are many limitations and inconsistencies in the reviewed studies, which obviously reduced the reproducibility and reliability of the results. First, although most guides (including ADA and WHO) recommended to use the new consensus version of criterion (IADPSG 2010) [15] for GDM diagnosis, a few guides (including ACOG and ADA) also supported to use alternative criteria such as Carpenter-Coustan 1982 [63] and NDDG 1979 [64]. Thus, various versions of diagnostic criteria were used in these studies. Some of the key parameters, such as the application of an additional test, dosage of a glucose load, thresholds, and number of abnormal indicators for diagnosis, are different among these criteria (Table 2), which causes the pathological backgrounds of GDM groups to be different. Moreover, there are currently no specific standards to further classify GDM into subgroups. However, there is an increasing need to classify GDM patients into different degrees of severity or to identify patients for different treatments. Only one reviewed study divided GDM patients into two groups according to the glucose concentrations of 2 h OGTT [29]. And the GDM groups of many studies contain patients treated with dietary control or insulin, which may increase sample heterogeneity and decrease the power to identify reliable DE proteins.

In addition, other factors, such as the exclusion criteria, treatment strategies, and clinical characteristics (both maternal and fetal), also affect the constitutive portions of enrolled subjects. Many risk factors (including age, geography, history of diabetes, and BMI) are known to be associated with GDM [65]. Thus, it is also important to consider matchingsubjects for these factors between disease and normal groups. However, clinical characteristics such as gestational age, BMI, or fetal birth weight were found to be significantly different between GDM and NGT in several reviewed studies, which make it ambiguous to interpret the results. In addition, only one of the reviewed studies clearly described the ethnicity of the enrolled pregnant women. However, both maternal and paternal races were found to be associated with different rates of GDM [66]. Asians and Hispanics were found to have higher rates of GDM compared to whites and African Americans. With the increase of global migration and mixed race [67], it is suggested to adjust the race factor for multiracial samples.

### 3.2. Sample Size and Sample Processing Issues

When comparing different studies using the same method and sample type, .there also exist great differences. For example, only two DE proteins in the placenta were the same in two studies using similar gel-based proteomic methods [28, 36]. This may due to complex factors including but not limited to sample size, sample processing, and statistical method.

Sample size is an important issue that is easy to be overlooked. A small number of samples may lead to inaccurate results, while too many samples may also increase costs and waste of resources. Thus, the minimal sample size is usually required to be strictly calculated in clinical case-control and diagnostic test studies [68]. However, the considerations and formula for calculations are complex for most clinicians. Few studies actually applied a calculation of sample size according to a review of clinical diagnostic studies [69].

Sample processing can greatly affect the reproductivity of proteomic results in different laboratories. For example, placental tissue is a complex tissue with several heterogeneous cell types including cytotrophoblasts, syncytiotrophoblasts, mesenchymal cells, and fetal vascular cells. Although two reviewed studies claimed that the placental tissue was extensively washed to remove maternal and fetal blood, there are many more factors, such as delivery time, placental shape, placental weight, sampling region, sampling method, and storage condition, which are also needed to be carefully considered according to a proposed standard for placental sample collection [70]. Two reviewed studies used a peptidomic approach to identify endogenous peptides presented in peripheral serum or umbilical venous plasma. However, two different methods were used to enrich low-molecular-weight peptides: the weak cation exchange (WCX) magnetic bead kit and the molecular weight cutoff (MWCO) filter. The WCX magnetic bead purifies peptides based on physicochemical features, while the MWCO filter removes large proteins according to the molecular weight. Thus, the two methods may have different biases for peptide enrichment.

### 3.3. The Bottleneck of Statistical Methods

Finally, the statistical methods may be the bottleneck to identify bona fide targets. Most proteomic studies used the traditional *t* test or rank test methods to detect DE proteins [71]. However, these methods are usually only applicable to the data with a certain distribution and require large samples, which may be inefficient to dig out significant proteins in proteomic data. In addition, an empirical *P* value of 0.05 was usually used as a cutoff for statistical significance. For multiple comparisons of large dataset such as a microarray result, the calculation of adjusted *P* values was proposed to control the false discovery rate (FDR) [72]. However, due to the native features of proteomic technologies, applying this procedure may result in no significant proteins at all. For example, one study identifies 25 DE proteins based on the raw *P* values, while none of these proteins remained significant after the application of FDR corrections [42]. *P* values are usually used together with fold change to obtain more reliable results. It is known that the expression changes are underestimated due to the internal interferences of near isobaric ions in proteomic studies based on isobaric labeling [73]. Thus, low threshold values of 1.2 or 1.5 for fold change are also widely used in many studies.

## 4. Considerations and Recommendations for Future Studies

### 4.1. New Research Directions for Future Studies

The main application of proteomics in medicine is to develop biomarkers. The reviewed studies have already covered a broad range of research contents in terms of sampling time, sample types, and outcomes. As GDM is a progressive and complex complication, there are still some novel topics which are needed to be investigated in the future. First, a recent study established a pre-pregnancy model and found that a panel of four biomarkers, which was tested about 7 years before pregnancy, could be used to predict future GDM risks [74]. The result also indicated that GDM or prediabetes may already be developing long before pregnancy. It is known that interventions in early pregnancy have limited effects in preventing GDM. Thus, it will be of great significance to identify potential protein biomarkers before conception. Second, the glucose levels of some GDM patients could be controlled by diet and healthy lifestyle, while other patients need insulin or metformin therapy. It is clear that GDM could be further classified into subgroups. However, there is a lack of efficient methods and common standards in this field; protein biomarkers could also be developed to solve this problem. Third, only a few maternal complications or fetal outcomes have been investigated in these studies. However, many conditions and complications, such as overweight, obese, hypertension, and hyperlipidemia, are known to be associated with metabolic disorders. It is also interesting to explore the cross-talk between GDM and these conditions at the protein level. Finally, many studies also found that the treatment of GDM patients did not significantly reduce some of the maternal and fetal outcomes such as subsequent diabetes, metabolic syndrome, perinatal death, neonatal hypoglycemia, and childhood obesity [13, 75–77]. Thus, it is necessary to develop prognostic markers for postpartum and long-term outcomes in treated patients.

It is also needed to pay more attention to some new directions about the types of biomarkers. First, one of the reviewed studies found that only the specific glycosylated forms of PSG and fibronectin are differentially expressed between GDM and NGT samples [31]. Protein phosphorylation and acetylation are also known to play important roles in regulating the signaling pathways of insulin [78, 79]. Thus, it may be more efficient to search for disease markers in proteins with various postmodifications. Instead of identifying protein biomarkers, two reviewed studies focused on active peptides which are endogenously cleaved [29, 33]. Recently, more and more evidences showed that small open frames are hidden in traditional transcripts of mRNA, lncRNA, microRNA, and circRNA and could encode functional polypeptides [80–82]. Short and low-abundance peptides are usually difficult to identify using the shotgun proteomic strategy. Thus, it is anticipated that more peptidomic studies will be performed to identify known or novel functional peptides. Moreover, MS technology could also help to clarify the microheterogeneity issue for complex components. For example, a protein complex, which includes transthyretin (TTR), serum retinol-binding protein (RBP4), and retinol (ROH), was presented in peripheral blood and known to be associated with the development of insulin resistance [83]. The concentrations, expression ratios, and different types of isoforms (caused by various posttranslational modifications, amino acid change, and sequence truncation) are systematically analyzed to investigate the relationship between first-trimester maternal serum levels of the TTR-RBP4-ROH complex and the development of GDM. Specific ratios or variants are determined to be potential biomarkers for different types of GDM.

### 4.2. The Importance of Follow-Up Functional Studies

Besides identifying potential biomarkers for clinical usage, discovery proteomics also provided potential proteins that could explain the molecular mechanisms associated with the development of GDM and its outcomes. However, there are still two limitations for the initial discovery proteomics. First, only a list of protein names is generated; it is not known how these proteins interact with each other and what functional pathways are they involved in. Second, the DE proteins are prioritized based on statistical inferences. It is also not sure whether they have bona fide functions in the development of disease. Bioinformatics can partially solve the first problem, as it has been widely used in omic studies to translate the gene list into known functional categories [84, 85]. However, to clearly clarify these issues, functional proteomics and experimental studies are needed to verify the functions or explore the regulation relations of the identified DE proteins.

To avoid harming human bodies, various animal models and cell lines are used to mimic human diseases and to study the functions of individual genes. In addition, human xenograft and animal allograft are also widely used as preclinical models to study disease process and treatment effects [86]. Animal models for T2D were successfully developed with different levels of insulin resistance and beta cell dysfunction. It is also easy to establish rat models with gestational diabetes. However, human GDM is associated with complex maternal and fetal outcomes. Thus, it is still hard to fully reproduce the adverse outcomes in rat models [87]. Nevertheless, cell lines and animal models will promote to translate a protein list identified by proteomics into organized knowledge for the development of GDM.

## 5. Conclusions

In summary, GDM is a progressive and long-lasting pregnancy complication, it also has complex effects on both maternal and fetal health. The adoption of the new IADPSG guideline has greatly increased the number of diagnosis as well as the overall cost of health care. Thus, it is urgent to develop diagnostic biomarkers or to study the pathogenic mechanisms for the development of GDM and its outcomes. Over the decades, MS-based quantitative proteomic strategies have been shown to be efficient in discovering clinical biomarkers or pathogenic targets. In the present review, we systematically summarized the proteomic studies of GDM. Generally, the reviewed studies usually began with an initial discovery proteomic analysis to generate a list of significant proteins. Then, the protein list could be further used to identify potential biomarkers for diagnosis or key proteins for understanding the pathogenesis. For biomarker discovery, several proteins were shown to be effective in the diagnosis of GDM as measured by the AUC. Functional annotation of all the integrated significant proteins also showed that a small number of proteins are known to be involved in the regulation of insulin. KEGG enrichment analysis indicated that the protein list could also be used to identify novel signaling pathways indirectly associated with the development of GDM. Thus, these studies provided a valuable resource for the study of GDM.

Apart from these achievements, there are also some limitations to these proteomic studies. First, the reviewed studies are largely different in many aspects of the experimental design and procedure. Many factors such as diagnostic criteria, sample processing, proteomic method, and statistical method can greatly affect the identification of reproducible and reliable protein candidates. Thus, we suggest to adopt the same diagnostic criteria and standardized procedure to reduce variations in and between different studies, as well as to improve the identification of verifiable markers. Second, most of these studies stopped in the stage of initial discovery or performed simple experiments to verify the expression of several selected DE proteins using a small number of samples. A serial of validation or functional experiments must be performed to further evaluate the diagnostic value of biomarker candidates or to explore the molecular mechanism for the development of GDM. Then, the protein list generated in discovery proteomics can be translated into clinical or biological significances. Finally, we also pointed out some new directions for the identification of biomarkers in GDM, such as pre-pregnancy biomarkers, subgroup classification, treatment assessment, and complex biomarkers. On the other hand, we also discussed the importance of functional studies based on animal models and cell lines to investigate the molecular mechanisms involved in the pathogenesis of GDM and its outcomes. Thus, although the reviewed studies covered a broad range of research contents, there is still much work to do to verify the results or to address unexplored issues. We believe that the presented review provides constructive suggestions and recommendations for carrying out proteomic or follow-up studies of GDM or other pregnancy complications in the future.

## Figures and Tables

**Table 1:** Summary of diagnostic models for GDM.

Cite No.	Sample size (GDM vs. NGT)	Sample type	Sampling time (weeks)	Markers in a model	AUC
31	15 vs. 14	Serum	24-28	(1) Fibronectin-SNA and PSG-AAL	0.85
				(2) CRP, adiponectin, SHBG, ratio of hCG to placental lactogen, fibronectin-SNA, and PSG-AAL	0.97
37	20 vs. 20	Serum	12-16	APOE, F9, FGA, and IGFBP5	0.985
38	40 vs. 40	Urine	15-20	CD59 and IL1RA	0.906
42	105 vs. 105	Serum	8-13	(1) Vitronectin	0.625
				(2) Maternal age, history of diabetes, and vitronectin	0.806
47	25 vs. 25	Plasma	11-13	(1) TSP-4	0.94
				(2) CNDP1	0.98

SNA: Sambucus nigra lectin; PSG: pregnancy-specific glycoprotein; AAL: Aleuria aurantia lectin; CRP: C-reactive protein; SHBG: sex-hormone-binding globulin; hCG: human chorionic gonadotropin; APOE: apolipoprotein E; F9: coagulation factor IX; FGA: fibrinogen alpha chain; IGFBP5: insulin-like growth factor-binding protein 5; CD59: CD59 glycoprotein; IL1RA: interleukin-1 receptor antagonist protein; TSP-4: thrombospondin-4; CNDP1: beta-ala-his dipeptidase.

**Table 2:** Comparison of representative criteria for GDM diagnosis.

Criteria	First step	Glucose load	Thresholds (mmol/L)				Number of indicators
			FPG	1 h	2 h	3 h	
IADPSG 2010	NA	75 g	5.1	10	8.5	NA	≥1
WHO 1999	NA	75 g	7.0	NA	7.8	NA	≥1
ADIPS 1998	NA	75 g	5.5	NA	8	NA	≥1
C-C 1982	50 g GLT	100 g	5.3	10	8.6	7.8	≥2
NDDG 1979	50 g GLT	100 g	5.8	10.6	9.2	8.0	≥2

C-C: Carpenter-Coustan; NDDG: National Diabetes Data Group; ADIPS: Australian Diabetes in Pregnancy Society; NA: none; FPG: fasting plasma glucose; GLT: glucose load test.

## Supplementary Materials

Supplementary Materials Supplement Data 1: the detailed information for the reviewed literatures. Supplement Data 2: list of the integrated differentially expressed proteins. Supplement Data 3: list of enriched pathways in different sample groups. Supplement Data 4: list of enriched pathways in different sampling time.
